# Macular Findings Obtained by Spectral Domain Optical Coherence Tomography in Retinopathy of Prematurity

**DOI:** 10.1155/2014/468653

**Published:** 2014-12-03

**Authors:** Muhammet Kazim Erol, Ozdemir Ozdemir, Deniz Turgut Coban, Ahmet Burak Bilgin, Berna Dogan, Esin Sogutlu Sari, Devrim Toslak

**Affiliations:** ^1^Department of Ophthalmology, Antalya Training and Research Hospital, 07030 Antalya, Turkey; ^2^Department of Ophthalmology, Zekai Tahir Burak Women's Health Training and Research Hospital, 06110 Ankara, Turkey; ^3^Department of Ophthalmology, Faculty of Medicine, Akdeniz University, 07070 Antalya, Turkey; ^4^Department of Ophthalmology, Faculty of Medicine, Balıkesir University, 10145 Balıkesir, Turkey

## Abstract

*Purpose.* To examine the macular findings obtained with spectral domain optical coherence tomography (SD OCT) in infants with retinopathy of prematurity (ROP).* Materials and Methods.* The macular SD OCT images of 190 premature infants were analyzed. Data regarding central foveal thickness (CFT), cystoid macular edema (CME), and cyst grading were compared. The relationships of CFT with gestational age and birth weight were investigated.* Results.* The results were obtained from 358 eyes of 179 infants (81 females and 98 males) of a mean gestational age of 30.9 ± 2.7 weeks and a mean birth weight of 1609 ± 477 g. ROP was diagnosed in 126 eyes and CME in 139 eyes. A significantly greater percentage of eyes with ROP were found to have CME (54%) compared to eyes without ROP (31%; *P* = 0.001). The incidence of CME was 46.3% for stage 1 ROP, 57.1% for stage 2, and 87.5% for stage 3. There was a weakly inverse correlation between CFT, gestational age, and birth weight (*P* = 0.025, *r* = −0.227; *P* = 0.002, *r* = −0.182, resp., Spearman correlation test).* Conclusions.* High-quality SD OCT images can be obtained from premature infants using the iVue system. Severity and frequency of CME in premature infants increase as stage of ROP increases.

## 1. Introduction 

Retinopathy of prematurity (ROP) is a vasoproliferative disease of the retina, that is, one of the most common causes of infant and childhood blindness [1]. Fortunately, the visual impairment related to ROP is largely preventable with timely treatment [2] after its diagnosis by ophthalmoscopic examination [3, 4]. However, it has recently been reported that disagreement may arise between ophthalmologists regarding the existence of plus disease [5], indicating subjectivity in diagnosis based on indirect ophthalmoscopic examination. To address the medical and legal issues that may arise regarding diagnosis of ROP, ophthalmologists have begun using new imaging systems such as RetCam (Clarity, USA), a digital fundus camera or computerized image analysis [6–8].

Optical coherence tomography (OCT) is an imaging system used for diagnosis and monitoring of many retinal diseases that captures cross-sectional images from the retinal pigment epithelium and photoreceptor layer. While OCT may provide more objective findings than other modalities, it is difficult to use in the imaging of infants. Fortunately, difficulty regarding the fixation and positioning of infants for imaging has been largely resolved with the development of portable OCT devices [9]. There are many studies about using portable OCT for imaging infants' retinas [10–16].

Studies performed with spectral domain (SD) OCT in premature infants have demonstrated the presence of several macular changes, such as cystoid macular edema [10–14], retinoschisis, and preretinal structures [15, 16], which were not detected by clinical examinations. Nevertheless, use of SD OCT in premature infants has not become widespread. To examine the macular findings in infants with ROP, we evaluated the capture of macular data and measurement of central foveal thickness by SD OCT.

## 2. Materials and Methods 

The study was approved by the local ethics committee and performed in accordance with the ethical standards outlined in the Declaration of Helsinki. Informed consent was obtained by the parents or guardians of all participating subjects prior to their inclusion in the study. The macular SD OCT images of all of the 190 premature infants (103 males and 87 females) who had participated in the ROP screening program at our hospital were analyzed retrospectively between June 2013 and May 2014. Infants with anterior segment anomalies like congenital glaucoma or congenital cataract and with corneal edema and posterior segment anomalies like choroidal coloboma, morning glory anomaly, and oculocutaneous albinism were excluded from the study.

In all ROP examinations, after placement of a lid speculum, central and peripheral fundus were examined carefully with scleral indentation using an indirect video ophthalmoscope (Heine Optotechnik, Herrsching, Germany). Next, OCT images were obtained from the center of the fovea with a SD OCT (iVue, Optovue, Fremont, USA). iStand which is a rolling floor stand option for iVue was used for scanning in supine position. When mounted to iStand, patients are able to be scanned in various positions including the supine position. iVue obtains 5-micron axial resolution in tissue using an 800 nm light source. Cross line scan gives us one horizontal and one vertical 2 × 6 mm image with 2.048 A scans and 5 micron resolutions. The device uses image averaging (20 B scans) to decrease the signal-to-noise ratio [17]. This feature allows us to evaluate the choroidal thickness in some patients. In this study, infants were scanned with cross line pattern in supine position. During scanning, his/her hands and head were held by a nurse. At least three scans were performed within 10 min for every patient to ensure obtaining at least three images of sufficient quality, in accordance with the manufacturer's suggestion. Low quality images were not subjected to review.

During the OCT imaging, a pacifier dipped in dextrose 30% was used to calm the infants without using any anesthesia and a lubricant eye drop (Systane, Alcon Pharmaceuticals Ltd, Barcelona, Spain) was instilled for hydrating the cornea as necessary. ROP grading was performed according to the International Classification of Retinopathy of Prematurity [4]. Infants with Type I ROP (zone I, any stage with plus disease; zone I, stage 3 without plus disease; zone II, stages 2-3 with plus disease) were treated with laser photocoagulation and/or intravitreal ranibizumab injection. Infants were then examined at intervals of four weeks, regardless of whether retinal vascularization had been completed, until the cystoid macular edema had been resolved. The SD OCT sections closest to the fovea were graded according to the following scale.

**Figure 1:**
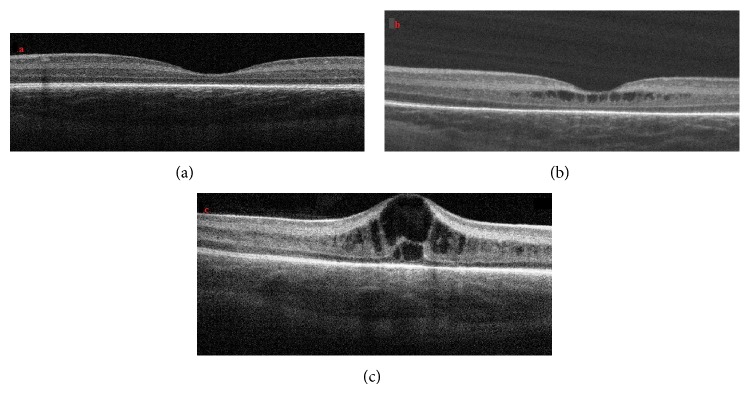
Examples of OCT images that demonstrate grade of cystoid macular edema. (a) Patient with no cystoid macular edema. (b) Patient with one or more cysts with no changes in the foveal contour. (c) One or more cysts with changes in the foveal contour.

 Grade 0: no cysts in the fovea (Figure 1(a)).
 Grade 1: one or more cysts with no changes in the foveal contour (Figure 1(b)).
 Grade 2: one or more cysts with changes in the foveal contour (Figure 1(c)).

To evaluate central foveal thickness, defined as the distance from the internal limiting membrane to the retina pigment epithelium (RPE), the OCT image at the steepest foveal excavation from the cross line scan was examined. When cystoid macular edema disruption of the foveal contour was present, measurement was performed at the point that protruded most greatly. Using the digital caliper tool built into SD OCT system, central foveal thickness was measured by two experienced observers who were blind to all group data.

Using the mean value of two measurements, statistical analysis was performed using analytical software (SPSS Inc., Chicago, IL, USA). Demographic characteristics and other data regarding the infants were analyzed statistically and reported in terms of the mean ± standard deviation (SD), frequency, and percentage. Data regarding central foveal thickness, cyst grading, and macular edema were compared using nonparametric tests (the Pearson chi-square test, Mann-Whitney *U* test, and Kruskal-Wallis *H* test). Differences reaching a significance level of *P* < 0.05 were considered statistically significant. The correlations between the central foveal thickness with gestational age and birth weight were calculated with Spearman's correlation test.

## 3. Results 

The results were obtained from 358 eyes of 179 infants (81 girls, 98 boys). The images of five infants that were of low quality, of 4 infants due to being obtained too far from the fovea, and of two infants in whom one-sided Peters' anomaly was detected were excluded from further examination. The mean gestational age was 30.9 ± 2.7 weeks (range 24–36 weeks), the mean birth weight was 1609 ± 477 g (range 640–2530 g), and the mean postmenstrual age at SD OCT imaging was 38.2 ± 3.9 weeks (Table 1).

A total 126 eyes had ROP, while 232 eyes had no ROP in visit. There was no significant differences in the mean postmenstrual age at the SD OCT imaging between the infants as a stage of ROP (*P* = 0.15, Kruskal-Wallis *H* test). Based on evaluation of the measurements, 139 eyes were diagnosed with macular edema and 219 eyes without macular edema. A greater percentage of eyes with ROP were found to have macular edema (53.9%) than eyes without ROP (30.5%), a difference that reached a level of statistical significance (*P* = 0.001; Mann-Whitney *U* test). The incidence of macular edema was found to be 46.3% for stage 1, 57.1% for stage 2, and 87.5% for stage 3. The presence of macular edema in eyes with ROP was increasing with rising of ROP stages (Table 2).

**Figure 2:**
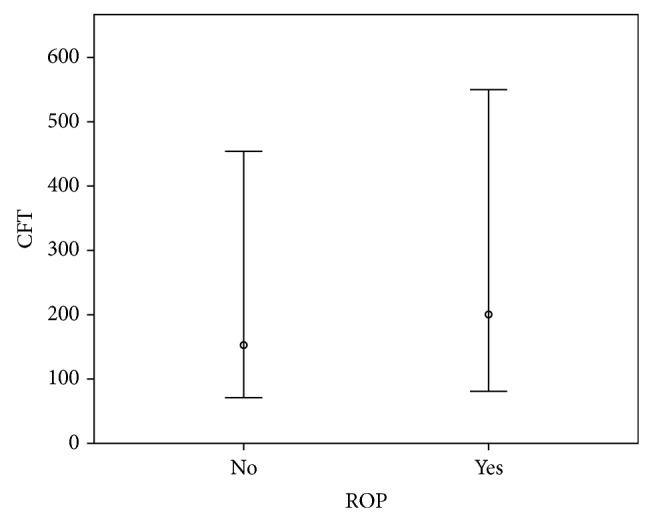
Comparisons of mean central foveal thickness (CFT) between the eyes with and without ROP. (ROP: retinopathy of prematurity, SD: standard deviation, and *P*: 0.001, Mann-Whitney *U* test).

**Figure 3:**
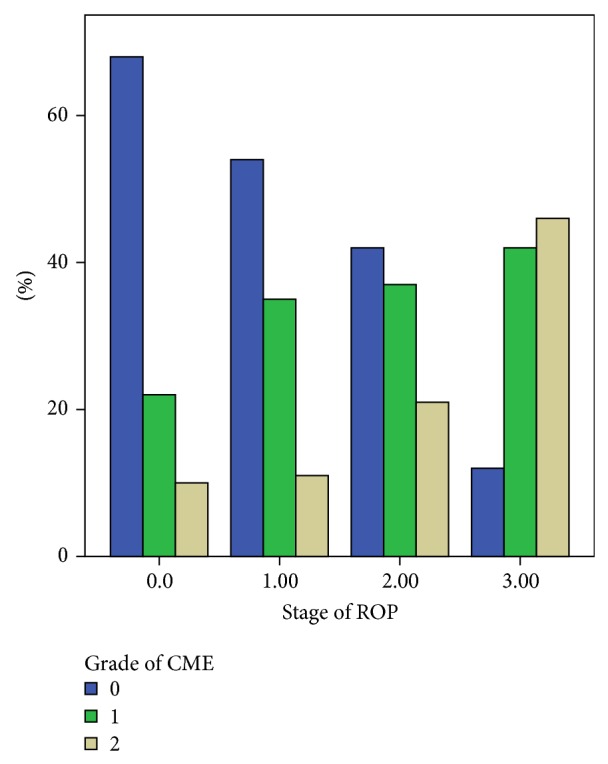
Comparisons of cystoid macular edema grading in eyes according to stage of ROP. (ROP: retinopathy of prematurity, *P*: 0.001 chi-square test).

Figure 2 shows the results of comparison of mean CFT between the eyes with and without ROP. The mean CFT of all eyes was 175.2 ± 106.5 *μ*m, while that of eyes without ROP was 163.3 ± 105.3 and that of eyes with ROP was 199.8 ± 97.3 *μ*m (stage 1: 192.2 ± 102.5 *μ*m, stage 2: 192.4 ± 70.7 *μ*m, and stage 3: 239.3 ± 69.7 *μ*m). The mean CFT was found to be significantly larger in eyes with ROP than eyes without ROP (*P* = 0.001, Mann-Whitney *U* test). Analysis of cyst grading based on OCT scanning revealed a significant difference between classifications of ROP stage, with cyst grade found to increase with increasing ROP stage (Figure 3).

**Figure 4:**
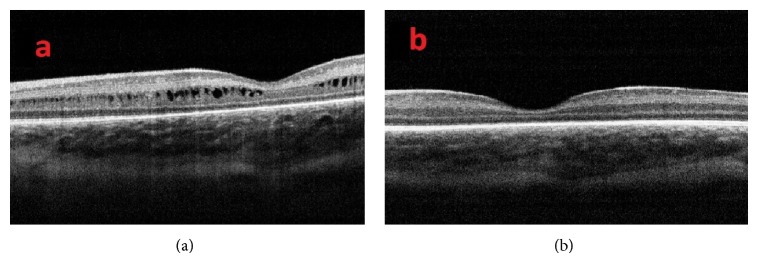
(a) OCT image of a patient with cystoid macular edema at 39-week postmenstrual age and (b) after resolution of cystoid macular edema six weeks later.

**Figure 5:**
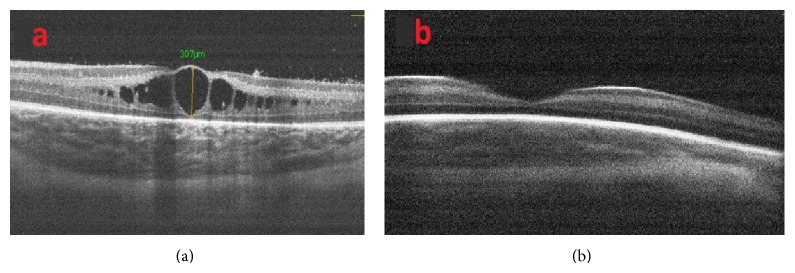
(a) OCT image of a premature infant with type 1 ROP (zone 2 stage 3 with plus disease) before intravitreal ranibizumab treatment and (b) two months after intravitreal ranibizumab treatment.

**Figure 6:**
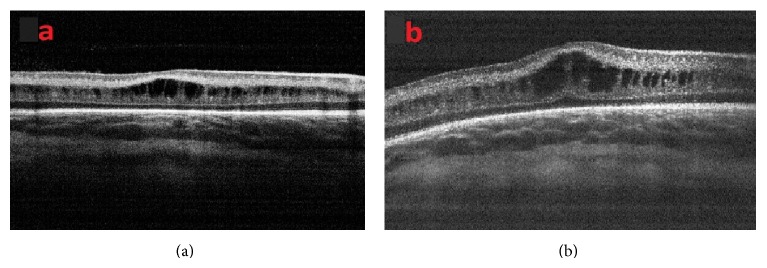
(a) OCT image of a premature infant with type 1 ROP (zone 2 stage 3 with plus disease) before intravitreal ranibizumab treatment and (b) one week after intravitreal ranibizumab treatment.

**Figure 7:**
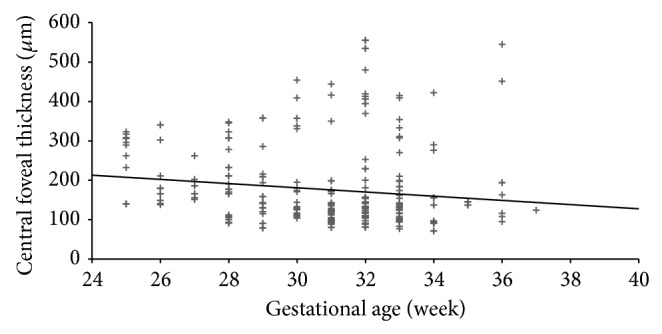
The correlation between central foveal thickness and gestational age (*P* = 0.025, *r* = −0.227) (Spearman correlation test).

**Figure 8:**
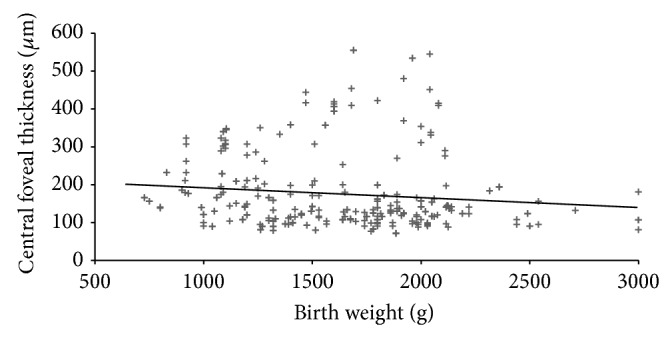
The correlation between central foveal thickness and birth weight (*P* = 0.002, *r* = −0.182) (Spearman correlation test).

At a mean postmenstrual age of 44.2 ± 3.5 weeks, regression of cystoid macular edema was observed in 71 eyes (Figure 4). Intravitreal ranibizumab injection was administered to five infants for treatment of Type 1 ROP at a mean postmenstrual age of 36.2 ± 1.9 weeks. CFT increased in three infants and did not increase in two infants within one week after intravitreal ranibizumab treatment (Figure 5). Of the two infants in whom CFT did not increase, cystoid macular edema resolved in one infant two months after intravitreal ranibizumab injection (Figure 6). At the mean 38.2 ± 3.9 weeks of postmenstrual age, there was a weakly inverse correlation between CFT and gestational age (*P* = 0.025, *r* = −0.227) (Figure 7) and between CFT and birth weight (*P* = 0.002, *r* = −0.182) (Figure 8).

## 4. Discussion 

SD OCT is widely used in the diagnosis and monitoring of many retinal diseases in adult patients. However, its use in the pediatric population has been limited due to the need to conduct scanning in the upright position. Nevertheless, Maldonado and Toth have identified two OCT devices, the Bioptigen and iVue systems, commercially available in the United States that are capable of obtaining SD OCT images while the patient is in the supine position [9]. Although only the Bioptigen has obtained Food and Drug Administration (FDA) approval for usage in children in the United States, the iVue can be obtained commercially in Turkey. As our hospital has obtained an iVue, we used it to examine ROP in premature infants in the current study. One advantage of using of the iVue is that it can be mounted to the iStand, which minimizes the production of imaging artifacts due to operator movement.

In a study with 31 prematurely born neonates imaged from 31 to 42 postmenstrual weeks (median birth weight was 825 g, median gestational age 26 weeks, and final ROP stage < 2), Maldonado et al. identified cystoid macular edema in 58% of patients [13]. In a study of 42 neonates, Dubis et al. identified cystoid macular edema in 56% of patients [14]. In the current study, the first to be conducted in a Turkish population, we detected cystoid macular edema in 38% of patients with a mean gestational age of 31 weeks and mean birth weight of 1609 g. Several factors may account for the discrepancy between our findings and those of other researchers. Infants that we examined were with higher gestational age and heavier birth weight than previous study. The discrepancy between our findings and those of Dubis et al. and Maldonado et al. could be attributed to the older age and greater body weight of the patients in the current study, whose initial imaging session was conducted at an older age compared to that of patients in previous studies. While the median age of the patient population in our study was similar to that in Vinekar et al.'s study, the use of different imaging devices may account for the differences found regarding cystoid macular edema. Specifically, Vinekar et al. reported a mean CFT of 156.9 ± 28.3 *μ*m, 206.5 ± 98.7 *μ*m, and 135.9 ± 17.6 *μ*m for stage 1, stage 2, and no ROP, respectively (age at OCT imaging for stage 2 patients: 37.18 ± 1.95 weeks of postmenstrual age) [11]. In contrast, we found a mean CFT of 192.2 ± 102.5 *μ*m, 192.4 ± 70.7 *μ*m, 239.3 ± 69.7 *μ*m, and 163.3 ± 105.3 *μ*m for stage 1, stage 2, stage 3, and no ROP, respectively (age at OCT imaging for stage 2 patients: 38.4 ± 4.3 weeks of postmenstrual age).

The etiology of cystoid macular edema observed in premature infants is not fully known. Maldonado et al. and Vinekar et al. have suggested that cystoid macular edema in prematurity may be related with vascular endothelial growth factor (VEGF), as is the cystoid macular edema observed in diabetic retinopathy and retinal vein occlusion [10, 11]. Indeed, it has been shown that growth factors, including VEGF, neuropilin, and semaphorin, have a role in the development of ROP fovea [18, 19]. However, upon observing development of cystoid macular edema after intravitreal bevacizumab injection in a premature infant and finding no correlation between stage of ROP and cystoid macular edema, Dubis et al. proposed that mechanical traction and neurohumoral factors other than VEGF could have a role in the development of cystoid macular edema in premature infants [14]. Having found that the density of RPE cells in premature infants is low, we propose that lower RPE cell density may contribute to formation of cystoid macular edema in premature infants [20]. Having observed a correlation between stage of ROP and cystoid macular edema in the current study, we also propose that the main factor in development of cystoid macular edema is VEGF. Nevertheless, we observed full retinal vascularization and regression of ROP in three infants who had cystoid macular edema (one of them with Grade 1, others with Grade 2). We also observed increase in cystoid macular edema one week after intravitreal ranibizumab injection in three of five infants to whom we administered this treatment.

Spontaneous regression of cystoid macular edema has been observed in previous studies [10–14]. Among them, Vinekar et al. observed regression of cystoid macular edema in 19 eyes of 10 babies at 52-week postmenstrual age [11], while Maldonado et al. observed the earliest regression of cystoid macular edema at 36 weeks and the latest at 43 weeks of postmenstrual age in nine babies [13]. In three patients in Dubis el al.'s study, cystoid macular edema was resolved at 52-week postmenstrual age in one and at postmenstrual age of 40 weeks in two [14]. We observed regression of cystoid macular edema in 71 eyes of 37 premature infants at a mean age of 44.2 ± 3.5 weeks of postmenstrual age and regression of cystoid macular edema after intravitreal ranibizumab injection in one infant at 44 weeks of postmenstrual age.

Primary treatment of ROP with intravitreal ranibizumab injection has been shown effective in decreasing cystoid macular edema secondary to diabetic retinopathy, retinal vein occlusion, and many other retinal diseases [21–24]. Nevertheless, Dubis et al. observed no regression of cystoid macular edema in four patients after intravitreal bevacizumab injection during the follow-up period [14]. However, in the small sample examined in our study, cystoid macular edema resolved in one patient two month after treatment, while cystoid macular edema grade increased in three patients one week after treatment and did not change in the month following treatment. Although bevacizumab and ranibizumab are used in treatment of ROP but not cystoid macular edema, their effect on the cystoid macular edema observed in ROP indicates that cystoid macular edema may be related with VEGF. Confirmation of the effects of ranibizumab treatment on the timing of cystoid macular edema regression now requires investigation of a larger sample over a longer follow-up period.

Among the many studies that have evaluated eyes with history of ROP [25–27], Park and Oh found that total retinal thickness and outer nuclear layer thickness in preterm infants were larger than those in term infants [25]. While, however, they and other researchers found that retinal thickness in preterm infants is not correlated with visual acuity [25–27], Parks et al. found an inverse correlation between birth week and retinal thickness in preterm infants. Similarly, we found an inverse correlation between birth week and central foveal thickness, although this correlation was weak. Although no association has been found between visual acuity and foveal structure, a multifocal electroretinography study observed that the most striking functional deficit was in the central region [28]. This finding leads us to speculate that this functional deficit may be associated with the cystoid macular edema observed during prematurity. Gaining greater understanding of the effect of cystoid macular edema on future visual function now requires follow-up of our patients over a longer period of time.

We faced several limitations in conducting this study that may have affected the results. First, as the study design was retrospective, we could not evaluate macula with SD OCT imaging on a weekly basis. Second, we used the iVue SD OCT system, which, to our knowledge, had not been previously used in premature infants. Third, although we believe we obtained high-quality images, we did not use the method for imaging premature infants defined by Maldonado et al. and other authors [29]. We instead used the method defined for adults, specifically the cross line mode rather than the retinal map mode, which allowed us to take one horizontal and one vertical high-quality image. Therefore, we may have failed to detect small cysts in other parts of the retina, and as the iVue system does not have a tracking system, repeated images could not pass in exactly the same place.

As a result, fovea of premature infants can be evaluated effectively by portable SD OCT devices. Transient cystoid macular edema is frequently seen in prematurity and the incidence of cystoid macular edema increases with the severity of ROP. Conducting a study with a larger sample over a longer follow-up period is necessary to evaluate the clinical significance of the cystoid macular edema observed in premature infants.

## Figures and Tables

**Table 1:** Demographic characteristics of infants.

Stage of ROP	Number of infants	Gender	Mean gestational age (week ±SD)	Mean birth weight (gram ±SD)	Mean postmenstrual age at the SD OCT imaging (week ±SD)
No ROP	116	52 F, 64 M	31.8 ± 1.8	1763 ± 446	38.5 ± 4.5
1	41	18 F, 23 M	29.3 ± 2.6	1368 ± 360	37.1 ± 2.5
2	14	8 F, 6 M	27.5 ± 2.1	1120 ± 328	38.4 ± 4.3
3	8	3 F, 5 M	26.7 ± 1.5	968 ± 146	38.5 ± 1.7

Total	179	81 F, 98 M	30.9 ± 2.7	1609 ± 477	38.2 ± 3.9

ROP: retinopathy of prematurity, SD: standard deviation, SD OCT: spectral domain optical coherence tomography.

**Table 2:** Comparisons of macular edema between the eyes with and without ROP.

ROP	Number of eyes	Macular edema	
		No	Yes
No	232	161 (69.5%)	71 (30.5%)
Yes	126	58 (46.1%)	68 (53.9%)
Stage 1	82	44 (53.7%)	38 (46.3%)
Stage 2	28	12 (42.9%)	16 (57.1%)
Stage 3	16	2 (12.5%)	14 (87.5%)

ROP: retinopathy of prematurity.
